# The Prognostic Prediction Value of Systemic Inflammation Score and the Development of a Nomogram for Patients With Surgically Treated Breast Cancer

**DOI:** 10.3389/fonc.2020.563731

**Published:** 2020-10-20

**Authors:** Zhang-Zan Huang, Xin Hua, Chen-Ge Song, Wen Xia, Xi-Wen Bi, Zhong-Yu Yuan, Zhen-Yu He, Jia-Jia Huang

**Affiliations:** Department of Medical Oncology, State Key Laboratory of Oncology in South China, Collaborative Innovation Center for Cancer Medicine, Sun Yat-sen University Cancer Center, Guangzhou, China

**Keywords:** SIS, breast cancer, surgery, nomogram, survival

## Abstract

**Background:** Systemic inflammation score (SIS) has been verified as a novel prognostic indicator in several cancer types. However, its prognostic value in breast cancer remains unknown. Furthermore, a nomogram based on SIS is yet to be constructed for breast cancer. We conducted this study to explore the association between SIS and prognosis of breast cancer, and to construct a good prognostic nomogram model.

**Methods:** A total of 1,180 breast cancer patients who underwent curative surgery between December 2010 and January 2013 were recruited. They were randomly assigned to the training set (*n* = 944) or the validation set (*n* = 236). All patient blood samples were collected within 1 week prior to operation. According to previous reports, SIS was calculated for all patients, who were then classified into two groups: high-SIS and low-SIS. The Kaplan–Meier method was employed for survival analyses, and univariate and multivariate analyses (Cox proportional hazards regression model) were used for prognostic assessment. A nomogram was constructed based on the results of multivariate analysis. Calibration curves and concordance index (C-index) were compiled to determine predictive and discriminatory capacity.

**Results:** In the training set, the median follow-up time was 6.07 years. Patients in the high-SIS group had an average OS time of 68.05 months, which is shorter than that of the low-SIS group (72.87 months; *P* = 0.033). Patients in the high-SIS group had average RFS and DMFS times of 56.04 and 54.46 months, respectively, which are shorter than those of the low-SIS group (60.85 and 59.47 months, respectively; *P* = 0.247 and *P* = 0.032). Univariate and multivariate analyses revealed SIS to be an independent prognostic factor for OS and DMFS time. The nomogram for the training set indicated OS and DMFS C-indexes of 0.794 (95% CI, 0.772–0.816) and 0.712 (95% CI, 0.684–0.740), respectively. In the validation set, the OS and DMFS C-indexes were 0.889 (95% CI, 0.845–0.933) and 0.696 (95%. CI, 0.611–0.781), respectively.

**Conclusions:** SIS was confirmed as an independent prognostic predictor among patients with breast cancer who had undergone surgery with curative intent. Higher preoperative SIS may indicate higher risk of metastasis and shorter overall survival time. The prognostic nomogram based on SIS was dependable for breast cancer patients who underwent curative surgery.

## Introduction

Breast cancer is the most common type of cancer in females, with the highest morbidity and mortality rates among all carcinomas in females (1). The diagnosis and management of breast cancer has advanced significantly, yet breast cancer remains a significant threat to female health. Surgery is generally regarded as the optimal therapeutic option when circumstances permit (2). According to estrogen receptor (ER), progesterone receptor (PR), and human epidermal growth factor receptor 2 (HER-2) status, breast cancer is divided into different subtypes (3). Each subtype has an associated appropriate postoperative therapy regime, involving chemotherapy, radiotherapy, endocrine therapy or targeted therapy (4, 5). Although a set of standard procedures has been implemented in the clinic, the death rate remains high, and recurrence and metastasis continue to occur. Therefore, the identification of novel prognostic indicators is essential to identify patients with a higher risk of recurrence and metastasis, so that appropriate treatment can be planned in advance. Various prognostic models are widely used for postoperative breast cancer patients, including the TNM staging system (6). However, due variation among individuals, accurate prediction of prognosis remains a challenge. A desirable prognostic predictor is one that considers individualized conditions.

Cancer-related inflammation is regarded as a hallmark of cancer (7, 8). In cancer patients, the systemic inflammatory response induces changes of the peripheral blood and plays a vital role in cancer pathogenesis and progression (9). Thus, several prognostic biomarkers associated with the systemic inflammatory response have been reported in various types of cancer, including neutrophil-to-lymphocyte ratio (NLR), platelet-to-lymphocyte ratio (PLR), and lymphocyte-to-monocyte ratio (LMR) (10–13). However, there exists no generally accepted thresholds for peripheral blood-based biomarkers or inflammatory scoring systems. There is a growing consensus that finding a simple, personalized, integrated scoring system based on inflammation is desirable. Recently, a novel systemic inflammatory scoring system, named systemic inflammation score (SIS), had attracted attention. SIS incorporates preoperative serum albumin levels and lymphocyte-to-monocyte ratio (LMR). The baseline LMR at diagnosis seemed to have unfavorable impact on the clinical outcomes in non-hematologic malignancies, including breast cancer, in the previous studies (14–17). Albumin, a classic nutrition index, was also considered as an inflammation related factor, and it was found to have prognostic significance in solid cancer in many studies (18–22). The novel inflammatory index, SIS, is quite promising in the recent studies to better reflect disease change and predict survival outcomes. In theory, SIS, combining LMR and albumin, could better reflect disease change and predict survival outcome. And this assumption has been reported to predict prognosis in several cancer types, including clear cell renal cell carcinoma, gastric cancer and colorectal cancer (23–25). There are no previous reports regarding the use of SIS in breast cancer. We hypothesize that SIS is an independent prognostic indicator in breast cancer.

The nomogram is a widely used medical statistical method, which integrates diverse prognostic and determinant variables to predict event probability (26–28). The purpose of this study is to investigate the association between SIS and other clinical characteristics, and the capacity of SIS as a prognostic predictor for postoperative patients with breast cancer. We present a nomogram based on SIS that provides a reliable prognostic prediction method for patients with breast cancer.

## Patients and Methods

### Patients

A total of 1,180 patients with breast cancer were enrolled in this study. All patients underwent surgical resection with curative intent at the Sun Yat-sen University Cancer Center (SYSUCC; Guangzhou, China) between December 2010 and January 2013. The inclusion criteria were as follows: (1) histologically confirmed breast cancer; (2) postoperative of resection with curative intent. The exclusion criteria were as follows: (1) synchronal malignancies; (2) ductal carcinoma *in situ*; (3) lack of associated laboratorial records or missing follow-up data; (4) prior receipt of neoadjuvant or adjuvant chemotherapy; (5) receipt of any medicine within 3 months prior surgery that could induce immune or inflammatory responses; (6) any chronic inflammatory disease (including autoimmune diseases). This study was performed in accordance with the declaration of Helsinki and was approved by the Research Ethics Committee of SYSUCC. All subjects enrolled provided their written informed consent.

### Sample Collection and Classification

All patient records were derived from SYSUCC. Blood samples were collected and analyzed within 1 week prior to surgery. Patients were classified into two groups based on median age. Tumors were staged according to the 8th AJCC (American Joint Committee on Cancer) Tumor-Node-Metastasis (TNM) staging system (6). The expression of ER, PR, HER2, Ki67 were scored using the St. Gallen criteria (3). Overall survival (OS) time was defined as the period from date of surgery to that of death or last follow-up. Recurrence-free survival (RFS) was defined as the period from the date of surgery to that of identification of first recurrence, death from any cause or last follow-up. Distant metastasis-free survival (DMFS) time was defined as the period from the date of surgery to that of identification of metastasis, death from any cause or last follow-up. The SIS score was calculated using two factors: serum albumin level and LMR. Patients with both hypoalbuminemia (<40 gL−1) and low LMR (<4.44) were assigned a score of 2; patients with either a decreased serum albumin level (<40 gL−1) or low LMR (<4.44) were assigned a score of 1; patients with a serum albumin level ≥ 40 gL−1 and LMR ≥ 4.44 were assigned a score of 0.

### Follow-Up

Patients were followed up from the day of operation. Long term follow-up was performed by outpatient examination or telephone interview for the first 2 years and every 6 months for the following 3 years. The date of last follow-up date was September 2019.

### Statistical Analysis

Statistical analyses were conducted using SPSS software (version 25.0; IBM Corp., Armonk, NY), GraphPad Prism (version 6.0; GraphPad, La Jolla, CA) or R software (“rms” package; version 5.1–0; Vanderbilt University, Nashville, TN). Survival curves were plotted using the Kaplan-Meier method, and significance was determined by the log-rank test. The associations between SIS and other key clinicopathological characteristics were analyzed by Chi-square test or Fisher's exact test. Simple and multivariate regression analyses were performed using the Cox proportional hazards model and multivariate regression analyses model for variables with *P* < 0.05 in the univariate analysis. Two-tailed *P*-values < 0.05 were considered to indicate statistically significant differences. A nomogram was created by R software using “rms” package and the nomograms of 3- and 5-year OS and DMFS times were developed. In this study, a validation group was used for internal validation. The concordance index (C-index) and calibration plots were calculated using OS and DMFS times both in training group and validation group. Calibration plots were generated to examine the performance characteristics of the predictive nomogram. The Harrell's Concordance index (C-index) was used to quantify the predictive accuracy (29), which ranges from 0.5 (no predictive power) to 1 (perfect prediction). All statistical tests were two-sided and were performed at a significance level of 0.05.

## Results

### Grouping by SIS Score

The cutoff of SIS was set at 1 and patients were grouped according to SIS score: group0 (low-SIS), group1 and group2 (high-SIS).

### Clinicopathological Features and SIS Scores of the Training Set

All patient characteristics are listed in Tables 1, 2. The median follow-up time was 6.07 years and the median age at surgery was 48 years old. A total of 822 (87.1%) patients had invasive ductal carcinoma, and 122 (12.9%) patients had other types of carcinoma. There were 209 (22.1%), 513 (54.4%) and 222 (23.5%) patients in stage 1, 2, and 3, respectively, according to the TNM staging system. A total of 205 (21.7%) patients were diagnosed as Luminal A, 354 (37.5%) as Luminal B/HER2–, 119 (12.6%) as Luminal B/HER2+, 125 (13.2%) as HER2 Enriched, and 141 (14.9%) as Triple Negative. ER expression was positive in 663 (70.2%) patients, PR expression was positive in 585 (62.0%) patients and HER2 expression was positive in 285 (30.2%) patients. Ki67 status was positive in 631 (66.8%) patients and negative in 313 (33.2%) patients. According to the aforementioned SIS classification system, 649 (68.7%) patients were stratified to low-SIS, and 295 (31.3%) patients to high-SIS. According to our stratification of SIS, we observed that high-SIS was associated with high T-stage.

**Table 1:** Clinicopathological patient characteristics.

**Characteristic**	**Training set**	**Validation set**
	**(*N* = 944)**	**(*N* = 236)**
**Age (years)**		
≤48	483 (51.2%)	117 (49.6%)
>48	461 (48.8%)	119 (50.4%)
**Histological type**		
Invasive ductal carcinoma	822 (87.1%)	182 (77.1%)
Others	122 (12.9%)	54 (22.9%)
**Tumor grade**		
1	27 (2.9%)	6 (2.5%)
2	650 (68.9%)	168 (71.2%)
3	191 (20.2%)	47 (19.9%)
Unknown	76 (8.0%)	15 (6.4%)
**T stage**		
0	2 (0.2%)	0 (0.0%)
1	313 (33.2%)	101 (42.8%)
2	552 (58.5%)	109 (46.2%)
3	43 (4.6%)	10 (4.2%)
4	34 (3.6%)	16 (6.8%)
**N stage**		
0	482 (51.1%)	124 (52.5%)
1	260 (27.5%)	63 (26.7%)
2	121 (12.8%)	28 (11.9%)
3	81 (8.6%)	21 (8.9%)
**Clinical stage**		
1	209 (22.1%)	67 (28.4%)
2	513 (54.4%)	109 (46.2%)
3	222 (23.5%)	60 (25.4%)
**Molecular subtype**		
Luminal A	205 (21.7%)	61 (25.8%)
Luminal B/HER2-	354 (37.5%)	65 (27.6%)
Luminal B/HER2+	119 (12.6%)	31 (13.1%)
HER2 Enriched	125 (13.2%)	42 (17.8%)
Triple Negative	141 (14.9%)	37 (15.7%)
**ER**		
Negative	281 (29.8%)	88 (37.3%)
Positive	663 (70.2%)	148 (62.7%)
**PR**		
Negative	359 (38.0%)	92 (39.0%)
Positive	585 (62.0%)	144 (61.0%)
**HER2**		
Negative	659 (69.8%)	159 (67.4%)
Positive	285 (30.2%)	77 (32.6%)
**Ki67**		
Negative	313 (33.2%)	90 (38.1%)
Positive	631 (66.8%)	146 (61.9%)
**Adjuvant chemotherapy**		
Yes	787 (83.4%)	174 (73.4%)
No	157 (16.6%)	62 (26.3%)
**Endocrine therapy**		
Yes	661 (70.0%)	146 (61.8%)
No	281 (29.8%)	90 (38.2%)
Unknow	2 (0.2%)	
**Radiotherapy**		
Yes	246 (26.1%)	76 (32.3%)
No	698 (73.9%)	160 (67.8%)
**Target therapy**		
Yes	61 (6.5%)	23 (9.7%)
No	883 (93.5%)	213 (90.3%)
**SIS-group**		
<1	649 (68.8%)	151 (63.9%)
≥1	295 (31.3%)	85 (36.1%)

*ER, estrogen receptor; PR, progesterone receptor; HER2, human epidermal growth factor receptor-2; SIS, Systemic inflammation score*.

**Table 2:** The relationship between SIS group and clinicopathological characteristics in the training set.

**Characteristic**	**Total (*N* = 944)**	**Systemic inflammation score**		***P*-value**
		**Low (0)**	**High (1,2)**	
**Age (years)**				
≤48	483 (51.2%)	327 (34.6%)	156 (16.6%)	0.477
>48	461 (48.8%)	322 (34.1%)	139 (14.7%)	
**Histological type**				
Invasive ductal carcinoma	822 (87.1%)	562 (59.6%)	260 (27.5%)	0.513
Others	122 (12.9%)	87 (9.2%)	35 (3.7%)	
**Tumor grade**				
1	27 (2.9%)	17 (1.8%)	10 (1.1%)	0.151
2	650 (68.9%)	453 (48.0%)	197 (20.9%)	
3	191 (20.2%)	121 (12.8%)	70 (7.4%)	
Unknown	76 (8.0%)	58 (6.1%)	18 (1.9%)	
**T stage**				
0	2 (0.1%)	0	2 (0.1%)	0.006
1	313 (33.2%)	203 (21.5%)	110 (11.7%)	
2	552 (58.5%)	401 (42.5%)	151 (16.0%)	
3	43 (4.6%)	26 (2.8%)	17 (1.8%)	
4	34 (3.6%)	19 (2.0%)	15 (1.6%)	
**N stage**				
0	482 (51.1%)	330 (35.0%)	152 (16.1%)	0.492
1	260 (27.5%)	187 (19.8%)	73 (7.7%)	
2	121 (12.8%)	80 (8.5%)	41 (4.3%)	
3	81 (8.6%)	52 (5.5%)	29 (3.1%)	
**Clinical stage**				
1	209 (22.1%)	139 (14.7%)	70 (7.4%)	0.117
2	513 (54.4%)	367 (38.9%)	146 (15.5%)	
3	222 (23.5%)	143 (15.1%)	79 (8.4%)	
**Molecular subtype**				0.379
Luminal A	205 (21.7%)	135 (14.3%)	70 (7.4%)	
Luminal B/HER2-	354 (37.5%)	241 (25.5%)	113 (12.0%)	
Luminal B/HER2+	119 (12.6%)	91 (9.6%)	28 (3.0%)	
HER2 Enriched	125 (13.2%)	86 (9.1%)	39 (4.1%)	
Triple Negative	141 (15.0%)	96 (10.2%)	45 (4.8%)	
**ER**				
Negative	281 (29.8%)	195 (20.7%)	86 (9.1%)	0.781
Positive	663 (70.2%)	454 (48.1%)	209 (22.1%)	
**PR**				
Negative	359 (38.0%)	246 (26.0%)	113 (12.0%)	0.906
Positive	585 (62.0%)	403 (42.7%)	182 (19.3%)	
**HER2**				
Negative	659 (69.8%)	446 (47.2%)	213 (22.6%)	0.280
Positive	285 (30.2%)	203 (21.5%)	82 (8.7%)	
**Ki67**				
Negative	313 (33.2%)	208 (22.1%)	105 (11.1%)	0.284
Positive	631 (66.8%)	441 (46.7%)	190 (20.1%)	
**Adjuvant chemotherapy**				
Yes	787 (83.4%)	550 (58.3%)	237 (25.1%)	0.092
No	157 (16.6%)	99 (10.5%)	58 (6.1%)	
**Endocrine therapy**				
Yes	502 (53.2%)	395 (38.1%)	143 (15.1%)	0.133
No	440 (46.6%)	289 (30.6%)	151 (16.0%)	
Unknown	2 (0.2%)	1 (0.1%)	1 (0.1%)	
**Radiotherapy**				
Yes	246 (26.1%)	156 (16.6%)	90 (9.5%)	0.036
No	698 (73.9%)	493 (53.2%)	205 (21.7%)	
**Target therapy**				
Yes	61 (6.5%)	45 (4.8%)	16 (1.7%)	0.382
No	883 (93.5%)	604 (64.0%)	279 (29.5%)	

*P < 0.05. ER, estrogen receptor; PR, progesterone receptor; HER2, human epidermal growth factor receptor-2*.

### Survival Outcomes and Predicted Prognosis Based on the SIS Scoring System in the Training Set

**Figure 1:**
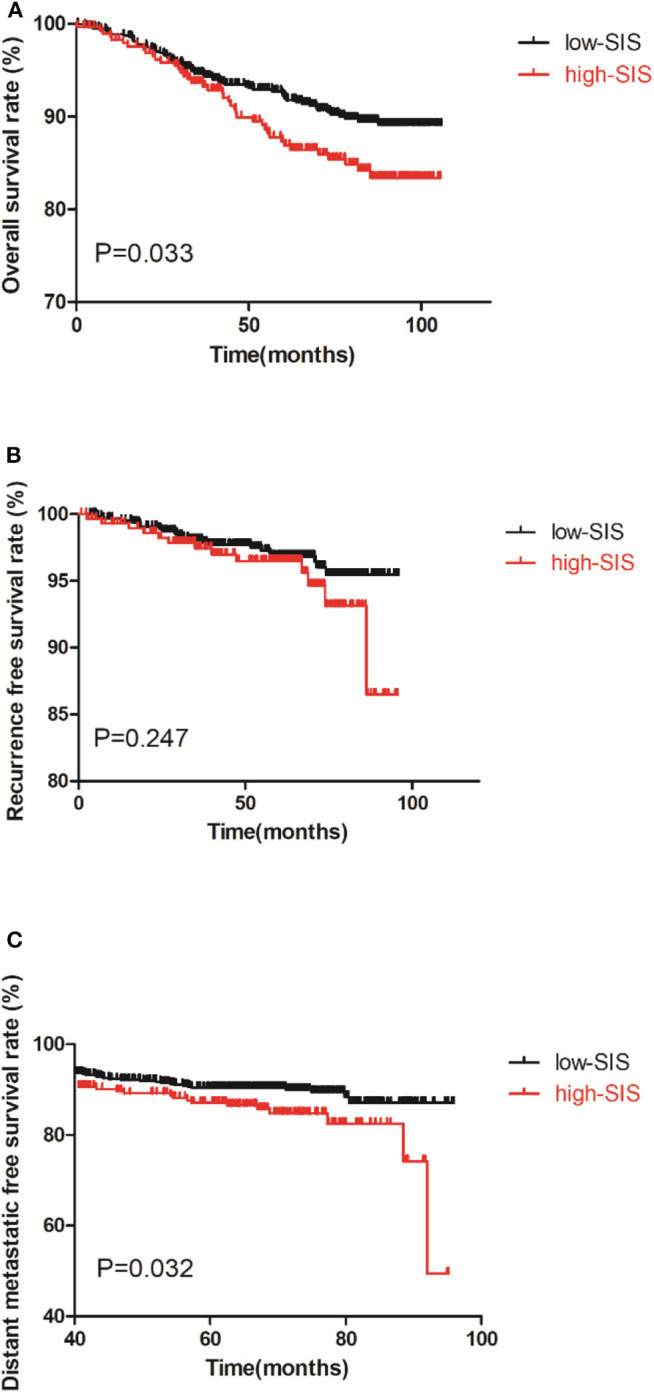
Kaplan-Meier survival curves of the training set of patients with breast cancer patients after surgery. **(A)** is the survival curves for OS, **(B)** is the survival curves for RFS, **(C)** is the survival curves for DMFS.

As shown in Figures 1A–C there was a correlation between high-SIS and poorer prognosis. The OS times of patients in the high-SIS and low-SIS groups were 68.05 and 72.87 months, respectively (*P* = 0.033). The RFS times of patients in the high-SIS and low-SIS groups were 56.04 and 60.85 months, respectively (*P* = 0.247). Finally, the DMFS times for patients in the high-SIS and low-SIS groups were 54.46 and 59.47 months, respectively (*P* = 0.032).

Univariate analysis showed that SIS group was associated with OS time (*P* = 0.034, Table 3) and DMFS time (*P* = 0.033, Table 4). Multivariate analysis revealed that SIS group was an independent factor influencing OS time (*P* = 0.035, Table 3) and DMFS time (*P* = 0.045, Table 4). Univariate and multivariate analyses for RFS were not performed.

**Table 3:** Univariate and multivariate analyses of overall survival in the training set.

**Characteristic**	**Univariate analysis**		**Multivariate Cox regression analysis**	
	**Hazard ratio (95% CI)**	***P*-value**	**Hazard ratio (95% CI)**	***P*-value**
Age (years)	1.276 (0.863–1.888)	0.222		
Histological type	0.271 (0.100–0.738)	0.011*	0.265 (0.091–0.765)	0.014*
Tumor grade	1.359 (1.009–1.830)	0.044*	0.936 (0.658–1.331)	0.714
T stage	1.843 (1.465–2.319)	0.000*	1.085 (0.842–1.400)	0.528
N stage	2.426 (2.036–2.891)	0.000*	2.471 (2.016–3.029)	0.000*
Clinical stage	4.200 (2.988–5.960)	0.000*		
Molecular Subtype	1.206 (1.052–1.383)	0.007*	1.243 (1.072–1.440)	0.004*
ER	0.671 (0.449–1.004)	0.052		
PR	0.640 (0.433–0.945)	0.025*		
HER2	1.745 (1.175–2.593)	0.006*		
Ki67	1.956 (1.210–3.163)	0.006*		
Adjuvant chemotherapy	1.596 (0.854–2.986)	0.143		
Endocrine Therapy	0.791 (0.536–1.167)	0.237		
Radiotherapy	1.836 (1.232–2.735)	0.003*	0.775 (0.505–1.189)	0.244
Target Therapy	0.851 (0.373–1.943)	0.702		
SIS group	1.538 (1.032–2.292)	0.034*	1.543 (1.031–2.310)	0.035*

* *P < 0.05. ER, estrogen receptor; PR, progesterone receptor; HER2, human epidermal growth factor receptor-2; SIS, Systemic inflammation score*.

**Table 4:** Univariate and multivariate analyses of distant metastatic free survival in the training set.

**Characteristic**	**Univariate analysis**		**Multivariate Cox regression analysis**	
	**Hazard ratio (95% CI)**	***P*-value**	**Hazard ratio (95% CI)**	***P*-value**
Age (years)	0.709 (0.472–1.064)	0.097		
Histological type	0.675 (0.340–1.340)	0.261		
Tumor grade	1.179 (0.886–1.567)	0.258		
T stage	1.469 (1.313–1.908)	0.004*	1.031 (0.779–1.364)	0.832
N stage	1.883 (1.581–2.242)	0.000*	1.783 (1.447–2.196)	0.000*
Clinical stage	2.691 (1.952–3.708)	0.000*		
Molecular subtype	0.933 (0.801–1.087)	0.374		
ER	1.252 (0.783–2.001)	0.348		
PR	0.934 (0.619–1.408)	0.744		
HER2	1.098 (0.714–1.690)	0.669		
Ki67	1.591 (1.003–2.524)	0.049*	1.480 (0.932–2.353)	0.097
Adjuvant chemotherapy	2.195 (1.065–4.526)	0.033*	1.579 (0.749–3.328)	0.230
Endocrine Therapy	1.403 (0.923–2.131)	0.113		
Radiotherapy	2.100 (1.403–3.143)	0.000*	1.101 (0.700–1.732)	0.678
Target Therapy	1.011 (0.467–2.189)	0.977		
SIS group	1.557 (1.035–2.341)	0.033*	1.521 (1.009–2.293)	0.045*

* *P < 0.05. ER, estrogen receptor; PR, progesterone receptor; HER2, human epidermal growth factor receptor-2; SIS, Systemic inflammation score*.

### Nomogram Construction and Validation

**Figure 2:**
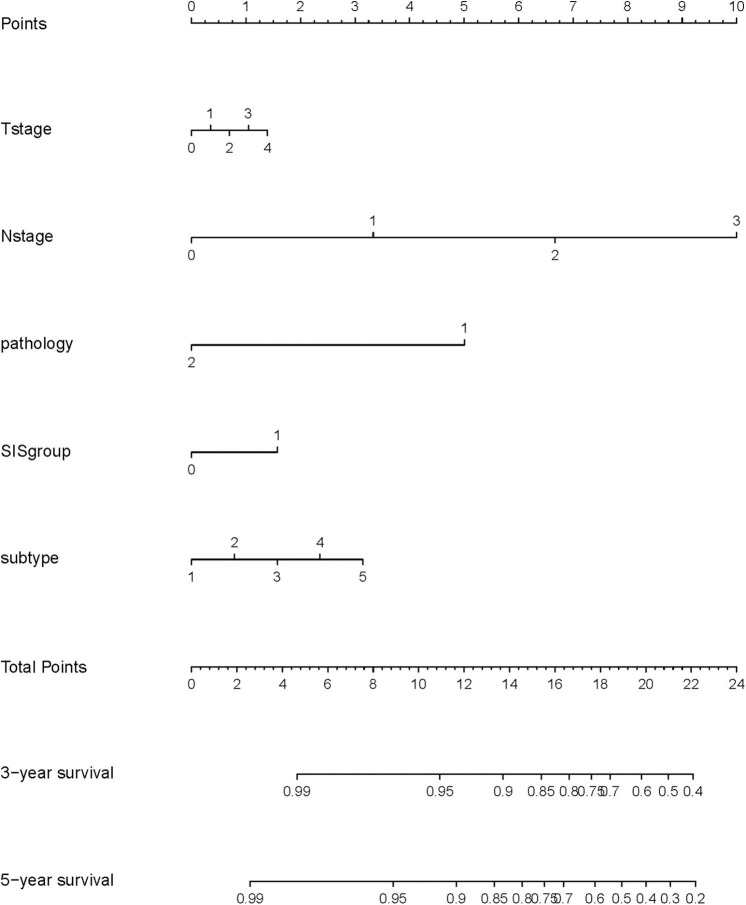
A nomogram predicting the 3- and 5-year overall survival of postoperative breast cancer patients. T stage: 0: T0; 1: T1; 2: T2; 3: T3; 4: T4. N stage: 0: N0; 1: N1; 2: N2; 3: N3. Pathology: 1: invasive ductal carcinoma; 2: others. SIS group: 0: low SIS group; 1: high SIS group. Subtype: 1: Luminal A; 2: Luminal B/HER2-; 3: Luminal B/HER2+; 4: HER2 Enriched; 5: Triple Negative.

**Figure 3:**
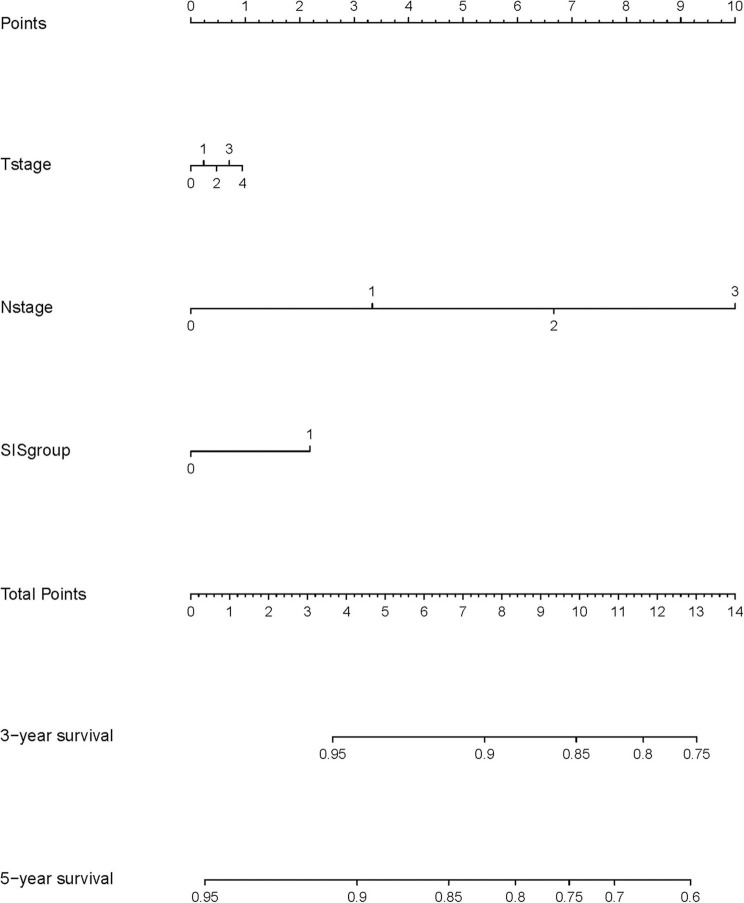
A nomogram predicting the 3- and 5-year distant metastatic free survival of postoperative breast cancer patients. T stage: 0: T0; 1: T1; 2: T2; 3: T3; 4: T4. N stage: 0: N0; 1: N1; 2: N2; 3: N3. SIS group: 0: low SIS group; 1: high SIS group.

A nomogram model was constructed based on the results of multivariate analysis. Independent prognostic indicators were integrated into the prediction of OS and DMFS times. With regard to OS time, these factors included N-stage, histological type, molecular subtype and SIS group (Figure 2), and for DMFS, these factors included N-stage and SIS group (Figure 3).

**Figure 4:**
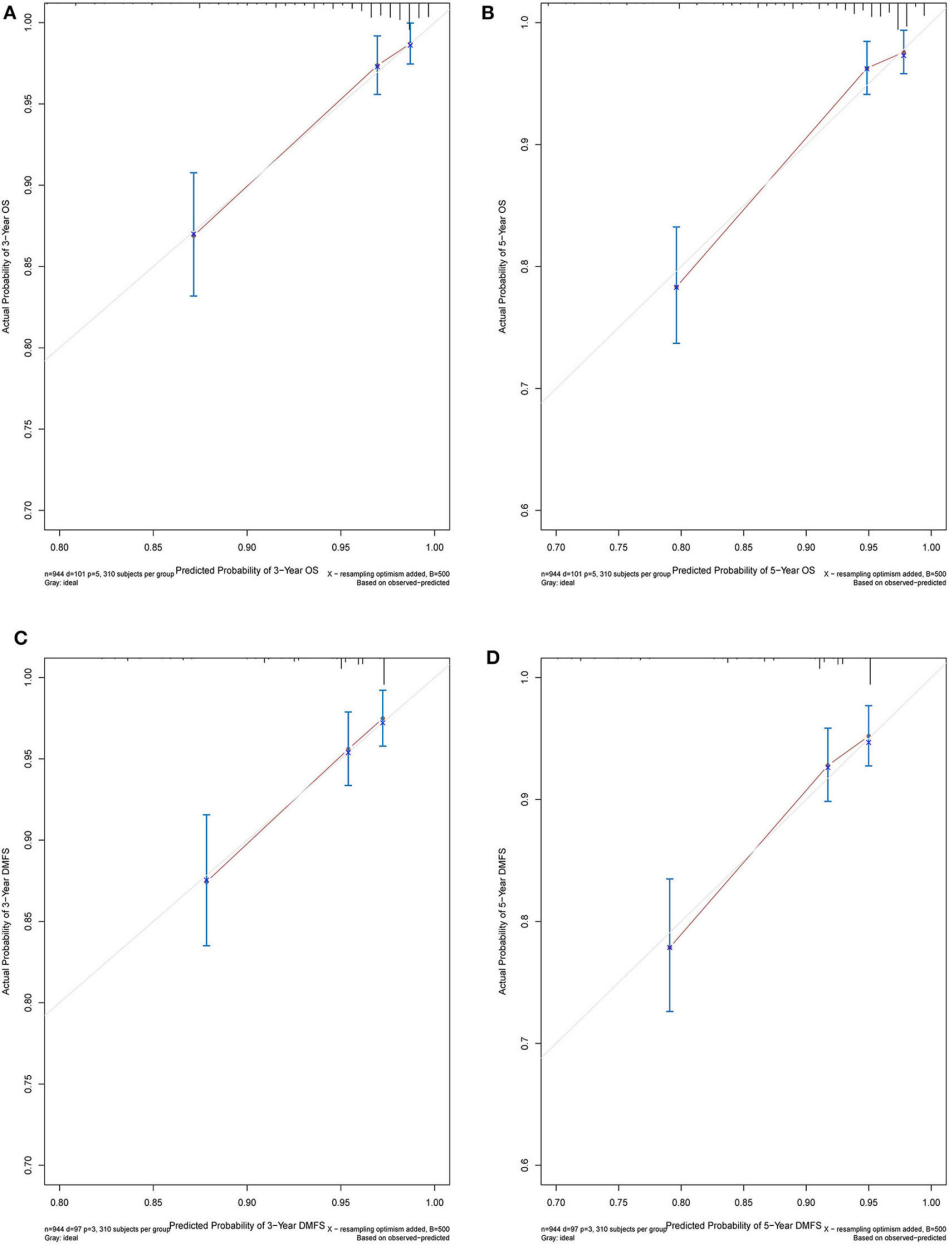
Nomogram model calibration curves of 3-year **(A)** and 5-year **(B)** overall survival, and 3-year **(C)** and 5-year **(D)** disease metastatic free survival in the training cohort.

**Figure 5:**
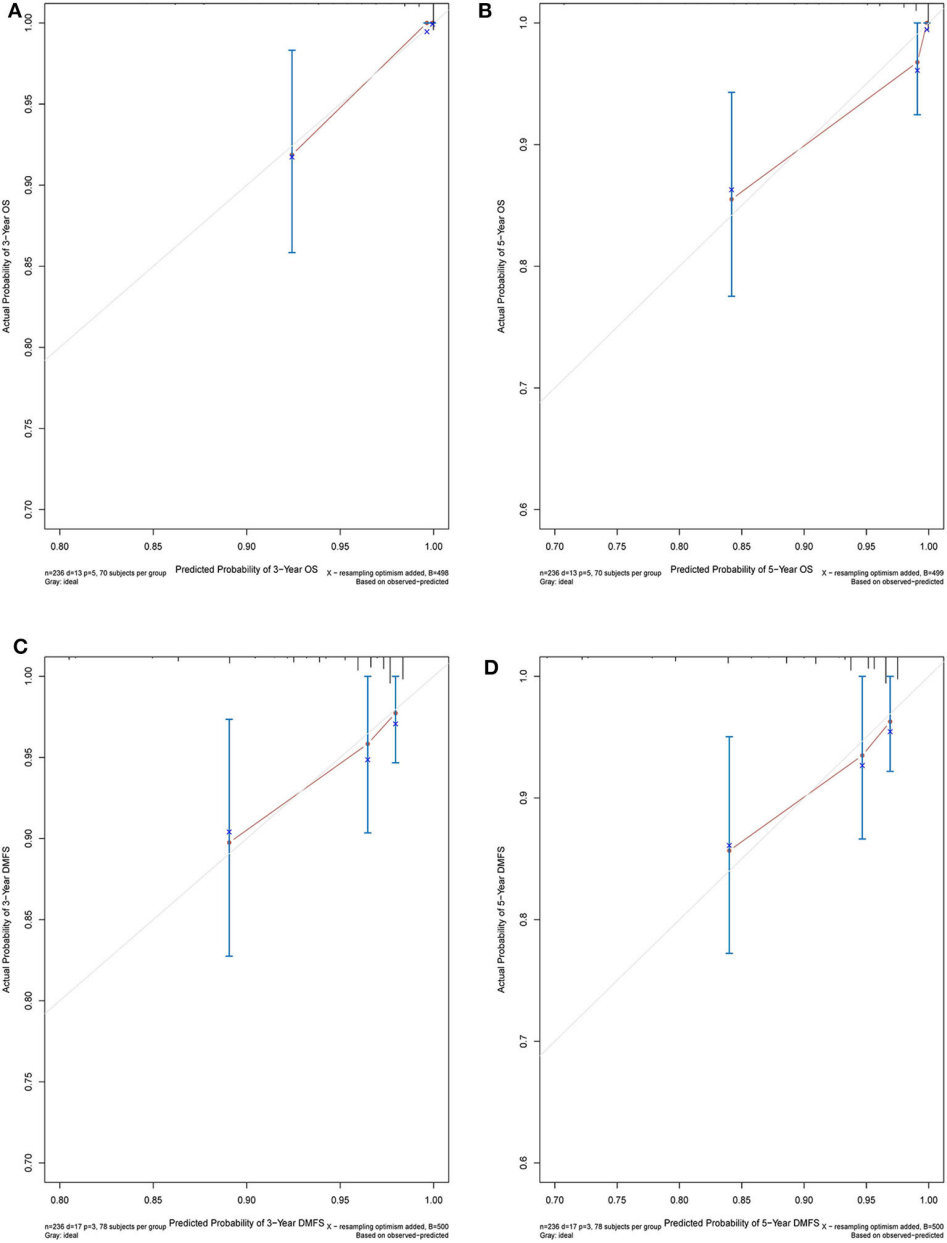
Nomogram model calibration curves of 3-year **(A)** and 5-year **(B)** overall survival, and 3-year **(C)** and 5-year **(D)** disease metastatic free survival in the validation cohort.

The C-index of the nomogram indicated good predictive accuracy for the survival of postoperative patients with breast cancer. The C-index for OS time was 0.794 (95% CI, 0.772–0.816) and for DMFS time, 0.712 (95% CI, 0.684–0.740). For the validation set, the C-index for OS time was 0.889 (95% CI, 0.845–0.933) and for DMFS time, 0.696 (95% CI, 0.611–0.781). The calibration curves indicated good consistency with actual observation in the use of this nomogram in prediction of 3- and 5- year OS and DMFS times in the training and validation cohorts (Figures 4, 5).

## Discussion

In this retrospective study, 994 patients were included in the training set and 236 patients in the validation set. We evaluated the association between SIS and clinicopathological characteristics, the association between SIS group and patient survival, and progressed to the construction of a nomogram prognosis prediction model. In the large training set of patients, we noted that high-SIS was positively associated with poorer prognosis of postoperative patients with breast cancer. High preoperative SIS was found to be indicative of shorter OS and DMFS times. Univariate and multivariate analyses revealed that high-SIS was an independent factor influencing OS and DMFS times of breast cancer patients. Cumulatively, these results demonstrate that high-SIS is strongly associated with tumor progression and shorter survival. Considering independent prognostic factors, we formed a calibrated nomogram model indicating favorable discrimination.

Virchow originally proposed a relationship between cancer and inflammation in 1863, and an increasing number of reports have since been published on this relationship, forming a general consensus that inflammation plays an important role in carcinogenesis (30). Inflammation substantially contributes to the development and progression of various types of cancer (31). Cancer-related inflammation contributes to the tumor microenvironment, therefore, in some types of cancer inflammation is present before a malignant change occurs (32, 33). Inflammation generates not only a cancer-promoting microenvironment, but also systemic changes that are favorable for cancer progression (8). In addition to local reactions, cancer-related inflammation also induces changes of the peripheral blood, including the count of lymphocyte and monocyte (34).

Lymphocytes inhibit tumor development through enhancing cancer immunosurveillance (35). Therefore, a decline in lymphocyte number implies poor tumor monitoring. Moreover, recent evidence has demonstrated that monocytes can be recruited to carcinoma tissues and differentiate into tumor-associated macrophages, which play key roles in stimulating angiogenesis, enhancing tumor cell migration and invasion, and suppressing anti-tumor immunity (36, 37). Therefore, increased monocyte numbers may be indicative of tumor progression. Given these facts, LMR is a good predictor of prognosis.

Serum albumin is a negative acute phase protein, routinely employed as a readout of patient nutritional status (38), but is also indicative of a sustained systemic inflammation response (39). SIS integrates LMR and serum albumin into a scoring system, easily accessible by routine blood test. SIS has been reported as a prognostic indicator in various cancer types, including clear-cell renal cell carcinoma, gastric cancer, colorectal cancer and lung cancer (23–25, 40). However, whether it is predictive of prognosis of patients with breast cancer remains unknown. The user-friendly nomogram has been applied in various oncology studies (28, 41–43), however, no previous studies have constructed a nomogram based on preoperative SIS. The present study aimed to determine the prognostic value of SIS in breast cancer patients after surgery.

It was observed that patients in the high-SIS group always had shorter survival than those in the low-SIS group (OS or DMFS time, all *P* < 0.05). By univariate analyses, SIS were shown to be associated with OS and DMFS time (all *P* < 0.05). By multivariate analysis, N-stage (*P* < 0.001), histological type (*P* = 0.011), molecular subtype (*P* = 0.005) and SIS group (*P* = 0.035) were identified as independent predictors of OS time, and N-stage (*P* < 0.001) and SIS (*P* = 0.045) were independent predictors of DMFS time. These results demonstrate that SIS can be used as a prognostic indicator for breast cancer patients after surgery, and the nomogram increases the credibility of this.

There were 101 deaths in the high SIS group. The cause of the death in the high SIS group was mainly related to disease progression (99/101). Besides, the baseline characteristics between the high and low SIS group were also compared. SIS at higher score was found to associate with aggressive behaviors, such as T stage (Table 2). In the multivariate analysis, SIS was shown to be a promising factor to predict the survival outcomes, independent of other clinical parameters. Therefore, the main reason of the worse mortality in the high SIS group was considered to be the progression of breast cancer. Nevertheless, more research is required to exclude the influence of these prognostic factors and to demonstrate that SIS is an independent prognostic factor of for surgically treated patients with breast cancer.

Clinically, SIS is an easy and inexpensive value to access. High preoperative SIS would indicate a higher risk of metastasis and shorter OS time, information which may assist clinicians in formulating more effective treatment plans.

There are some limitations in this study. Firstly, selection bias is difficult to avoid in retrospective studies. And the relationship and comparison between SIS and traditional TNM stage system and other inflammatory indicators, such as NLR, PLR, were not investigated. We concentrated on preoperative SIS status, but it would be beneficial to consider SIS over a disease/treatment time course. The following factors with the potential to affect prognosis were not considered: family history of breast cancer, histological grade, vascular invasion, number of lymphatic invasion events. Finally, our nomogram was validated internally, and external validation would be valuable.

In conclusion, we have demonstrated SIS to be an independent prognostic predictor of OS and DMFS time for patients with breast cancer patients after surgery with curative intent. High preoperative SIS is associated with poorer survival than low SIS. The nomogram derived from SIS grouping indicates satisfactory discrimination and consistency.

## Data Availability Statement

The datasets presented in this study can be found in online repositories. The names of the repository/repositories and accession number (s) can be found at: https://www.researchdata.org.cn/default.aspx, accession number RDDA2020002632.

## Ethics Statement

This study was performed in accordance with the declaration of Helsinki and was approved by the Research Ethics Committee of SYSUCC. All subjects enrolled provided their written informed consent.

## Author Contributions

Z-ZH, Z-YH, and J-JH contributed to conception and design of the study. XH organized the database. C-GS performed the statistical analysis. Z-ZH wrote the first draft of the manuscript. WX, X-WB, and Z-YY wrote sections of the manuscript. All authors contributed to the article and approved the submitted version.

## Conflict of Interest

The authors declare that the research was conducted in the absence of any commercial or financial relationships that could be construed as a potential conflict of interest.
